# Supplementary material for: What do Iranians value most when choosing a hospital? Evidence from a discrete choice experiment
Source: PLoS One. 2024 Oct 17;19(10):e0293241. doi: 10.1371/journal.pone.0293241 (PMC11486428; doi:10.1371/journal.pone.0293241)
Supplement: Attachment — Submitted filename: Response to Reviewers.docx [file pone.0293241.s003.docx]

Response to comment number 6

Reviewers' comments:

Reviewer's Responses to Questions

**Comments to the Author**

1. If the authors have adequately addressed your comments raised in a previous round of review and you feel that this manuscript is now acceptable for publication?

Reviewer #1: (No Response)

Reviewer #3: All comments have been addressed

2. Is the manuscript technically sound, and do the data support the conclusions?

Reviewer #1: Partly

Reviewer #3: Yes

3. Has the statistical analysis been performed appropriately and rigorously?

Reviewer #1: Yes

Reviewer #3: Yes

4. Have the authors made all data underlying the findings in their manuscript fully available?

Reviewer #1: Yes

Reviewer #3: Yes

5. Is the manuscript presented in an intelligible fashion and written in standard English?

Reviewer #1: Yes

Reviewer #3: Yes

6. Review Comments to the Author

**Reviewer #1:** Within the context of this study, a significant concern arises regarding the patient's limited ability to choose their preferred hospital. Instead, the doctor holds complete discretion in determining the hospital for medical services. In light of this, the question arises as to how we can effectively assess the patient's preferences in hospital selection.

Response to reviewer:

Dear reviewer,

Regarding your concern about the limited ability of patients to choose a hospital and the full competence of a physician to select a hospital, I must say that such a right does not exist for physicians in the health system of Iran. As we mentioned in our manuscript, in Iran, according to the Patient Rights Law, the patient has the freedom to choose their service provider and treatment center. On the other hand, due to the lack of a referral system in Iran, access to specialist and hospitals is freely available, and patients can choose their healthcare provider and center without any restrictions. Although the opinion of the treating physician is important, in such a context, the physician does not decide instead of the patient and does not have such a right under the law. The physician only provides advisory opinions, and the final decision to choose the treatment center is entirely the responsibility of the patient and the patient's family. It is also clear that factors such as medical costs, insurance coverage, geographical access, and similar issues can influence patient access freely. In this study, we have addressed these issues and their role in hospital selection by individuals and have shown that despite free access to hospitals, factors such as age, distance, waiting time, service quality, and other issues put patients' choice rights under scrutiny.

Another issue is that we have sought to investigate individual's preferences in this study, which hospitals they prefer to choose for treatment when they are ill. Therefore, the study results help health policymakers to take into account public preferences in redesigning the health system and building future hospitals.

**Reviewer #3:** (No Response)

7. PLOS authors have the option to publish the peer review history of their article ([what does this mean?](https://journals.plos.org/plosone/s/editorial-and-peer-review-process#loc-peer-review-history)). If published, this will include your full peer review and any attached files.

If you choose “no”, your identity will remain anonymous but your review may still be made public.

**Do you want your identity to be public for this peer review?** For information about this choice, including consent withdrawal, please see our [Privacy Policy](https://www.plos.org/privacy-policy).

Reviewer #1: No

Reviewer #3: No
